# From Solid Tumor to Hematologic Malignancy: A Case Report of Acute Promyelocytic Leukemia Following Breast Cancer Treatment

**DOI:** 10.7759/cureus.110169

**Published:** 2026-06-03

**Authors:** Ammarah Tahir, Natalia Ahmad, Bushra Ahsan, Hiba Asif, Maria Haider Khan

**Affiliations:** 1 Pathology and Laboratory Medicine, Shaukat Khanum Memorial Cancer Hospital and Research Centre, Lahore, PAK; 2 Hematology and Medical Oncology, Shaukat Khanum Memorial Cancer Hospital and Research Centre, Lahore, PAK

**Keywords:** acute promyelocytic leukemia, acute promyelocytic leukemia (apl), hormonal therapy, invasive ductal carcinoma of breast, post-cytotoxic therapy, solid tumor

## Abstract

Acute promyelocytic leukemia (APL) is a rare and aggressive subtype of acute myeloid leukemia (AML) that typically presents with bleeding tendencies and cytopenias. It is commonly associated with the PML::RARA fusion and, if not recognized early, can lead to severe coagulopathy and early mortality. Although post-cytotoxic therapy APL has been described following exposure to chemotherapy or radiation, its occurrence after breast cancer treatment, especially in patients managed primarily with hormonal therapy, is exceptionally rare. We are reporting a case of a 50-year-old female with estrogen receptor (ER)/progesterone receptor (PR)-positive, human epidermal growth factor receptor 2 (HER2)-negative, grade III invasive ductal carcinoma of the right breast (cT2N1M0), treated with neoadjuvant hormonal therapy (luteinizing hormone-releasing hormone (LHRH) analogs and letrozole) and zoledronic acid, followed by breast-conserving surgery, axillary dissection, and adjuvant radiotherapy. Post-treatment imaging showed no recurrence. One year later, she presented to the emergency department with epigastric and retrosternal pain, melena, dyspnea, and spontaneous bruising. ECG revealed T-wave inversions with elevated troponin I, raising the suspicion of myocardial infarction. Laboratory workup showed leukocytosis, anemia, thrombocytopenia, and 84% abnormal promyelocytes on peripheral smear. Fluorescence in situ hybridization (FISH) was positive for the PML::RARA fusion gene, confirming APL, supported by bone marrow biopsy and flow cytometry. She was initiated on all-trans retinoic acid (ATRA), idarubicin, allopurinol, and steroids due to a high leukocyte count. This case highlights post-cytotoxic therapy APL and its rare presentation following breast cancer treatment.

## Introduction

Acute leukemia (AL) is a rapidly developing malignancy of blood and bone marrow that results from an excess of primitive white blood cells identified as blasts [1]. Acute myeloid leukemia (AML) and acute lymphoblastic leukemia (ALL) are its two primary subtypes. AML results from the proliferation of myeloid cells in the blood, whereas ALL is caused by the proliferation of immature lymphoid cells [2]. AML is a genetically and clinically diverse hematologic malignancy that can occur in individuals of any age [3]. Acute promyelocytic leukemia (APL) represents a unique clinicopathologic subtype of AML and is defined by a reciprocal chromosomal translocation, t(15;17)(q24;q21), leading to the formation of the PML::RARA fusion gene [4,5]. According to the WHO classification of hematolymphoid neoplasms 2022, APL has been renamed APL with PML::RARA fusion. Acceptable alternate terminologies are "APL with t(15;17)" and "APL." This molecular abnormality interferes with normal myeloid maturation and plays a central role in disease pathogenesis [5]. Although treatment outcomes have improved substantially with targeted therapy, early death continues to pose a major challenge, largely attributable to profound coagulation abnormalities at presentation [5,6]. The disease predominantly occurs in younger patients, with secondary APL being a rare entity.

Post-cytotoxic therapy AML typically occurs in two patterns: one following alkylating agent exposure, often preceded by myelodysplasia and emerging after a latency of five to 10 years, often associated with chromosome 5 and 7 abnormalities; and another arising about two years after topoisomerase II inhibitor therapy, commonly associated with 11q23 rearrangements [7,8]. According to the WHO classification 2022, all such presentations are grouped under the umbrella "myeloid neoplasms post-cytotoxic therapy" (MN-pCTs). Post-cytotoxic therapy APL refers to cases developing after cytotoxic chemotherapy or radiotherapy. Although rare, its incidence is increasing, reflecting greater availability of cancer therapies and improved overall cancer survival, leading to an increasing population at risk [8]. The development of APL following hormonal and radiation therapy for a solid tumor is an exceedingly rare and clinically challenging occurrence. This case study highlights the rare occurrence of APL after receiving hormonal and radiation therapy for breast cancer, as well as the measures taken for its diagnosis and management.

## Case presentation

**Figure 1:**
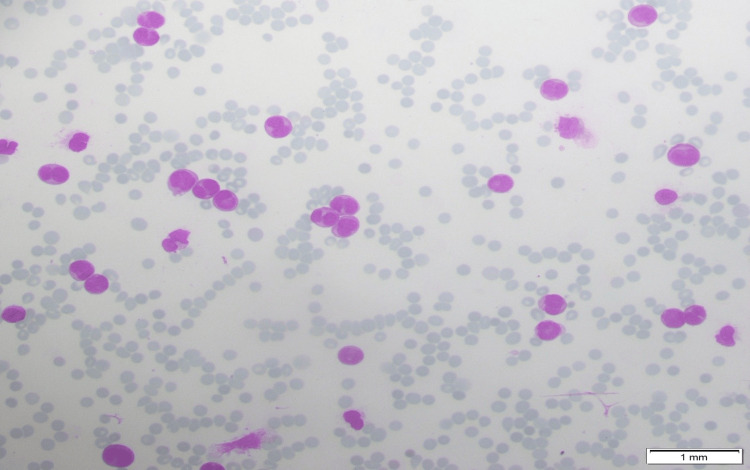
Peripheral smear showing abnormal promyelocytes

A 50-year-old female with a diagnosis of estrogen receptor (ER)/progesterone receptor (PR)-positive, human epidermal growth factor receptor 2 (HER2)-negative grade 3 invasive ductal carcinoma of the right breast with positive axillary lymph node involvement (pathological stage cT2N1M0) was offered neoadjuvant chemotherapy; however, she refused the treatment because of financial and religious considerations. She subsequently received neoadjuvant hormonal therapy comprising luteinizing hormone-releasing hormone (LHRH) analogs with letrozole, along with six-monthly doses of zoledronic acid (Zometa). She then underwent breast-conserving therapy, which included bi-localized wide local excision and sentinel lymph node biopsy, followed by axillary lymph node dissection due to a positive sentinel node. She subsequently received adjuvant radiotherapy to the right breast and right supraclavicular fossa. She completed her treatment with a post-treatment CT scan of the chest, abdomen, and pelvis, showing no evidence of disease recurrence. Exactly one year later, the patient presented to the emergency department with epigastric and retrosternal pain, vomiting, a history of melena and constipation, progressive dyspnea on minimal exertion, and spontaneous bruising. On physical examination, pallor and bruises over the bilateral arms were noted, with no lymphadenopathy or organomegaly. An ECG showed T-wave inversions and elevated troponin I (>113.56 ng/L), suggestive of a likely type II myocardial infarction. Cardiology consultation was obtained. The consulting cardiologist recommended initiation of statin therapy. Echocardiography showed a normal ejection fraction. Given the clinical picture suggestive of a type II myocardial infarction, management of the underlying precipitating cause and follow-up was advised, with no indication for dual antiplatelet therapy or invasive cardiac intervention at that stage. Symptomatic treatment, including analgesics, was administered. Initial laboratory evaluation was performed, and the complete blood count revealed leukocytosis, anemia, and thrombocytopenia (Table 1). Peripheral blood smear showed 84% blasts/abnormal promyelocytes (Figure 1). Patient was advised urgent fluorescence in situ hybridization (FISH) for PML-RARA, bone marrow biopsy, flow cytometry, and coagulation profile with suspicion of APL. 

**Table 1:** Complete blood count parameters

Complete Blood Count Parameters	Results	Reference Range
Hemoglobin (Hb)	5.8 g/dL	11-14.4 g/dL
Total leukocyte count (TLC)	86.06x10^3^/µl	4.52-10.93 x10^3^/µl
Platelet count	12 x10^3^/µl	150-450 x10^3^/µl

**Figure 2:**
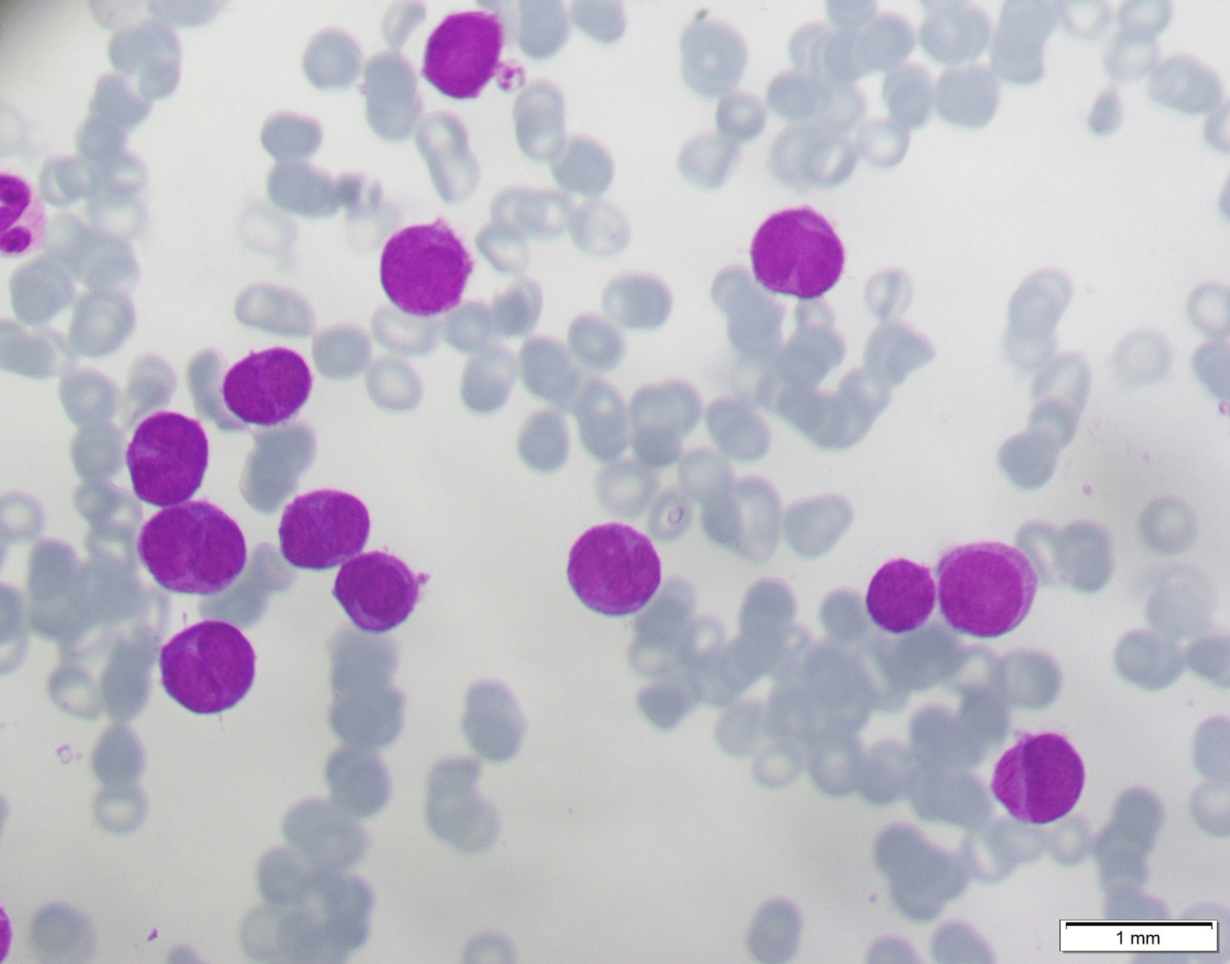
Bone marrow aspirate showing abnormal promyelocytes

**Figure 3:**
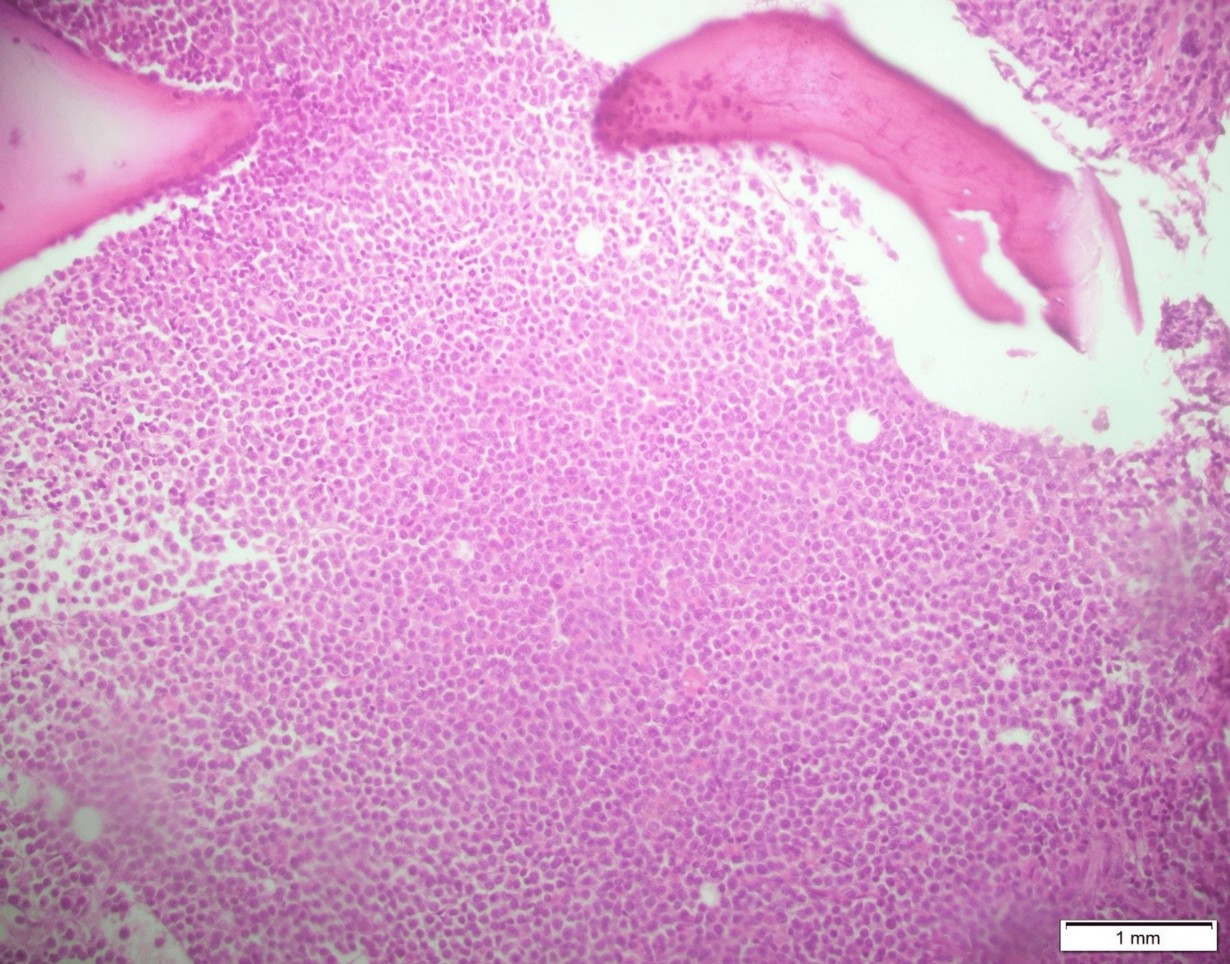
Bone marrow trephine

Bone marrow aspirate showed cellular particles and trails with all three cell lines (erythropoiesis, myelopoiesis, and megakaryopoiesis) were reduced. However, 93% of blasts/abnormal promyelocytes were identified in the bone marrow aspirate, which were medium to large in size, having a moderate amount of granular cytoplasm. Many of these cells exhibit nuclear folding. A few of these cells showed multiple Auer rods (Faggot cells) (Figure 2). Bone marrow trephine biopsy showed diffuse infiltration with abnormal promyelocytes (Figure 3). 

**Figure 4:**
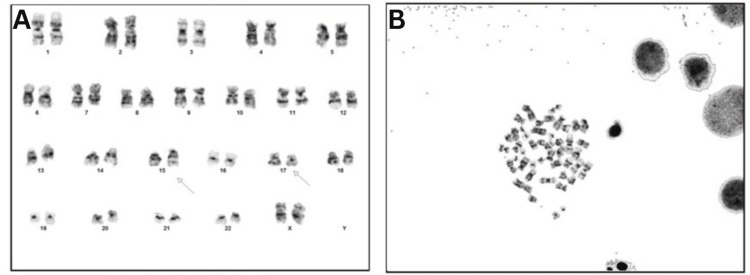
Conventional cytogenetic analysis of bone marrow metaphase cells A: karyogram showing 46,XX,t(15;17)(q24;q21); B: metaphase spread demonstrating the t(15;17) translocation, consistent with PML::RARA rearranged acute promyelocytic leukemia.

FISH for PML-RARA t (15;17) (q24; q21) on bone marrow aspirate revealed PML-RARA translocation in 86% of cells within 24 hours of sample submission, which confirms the diagnosis of APL (Figure 4).

**Figure 5:**
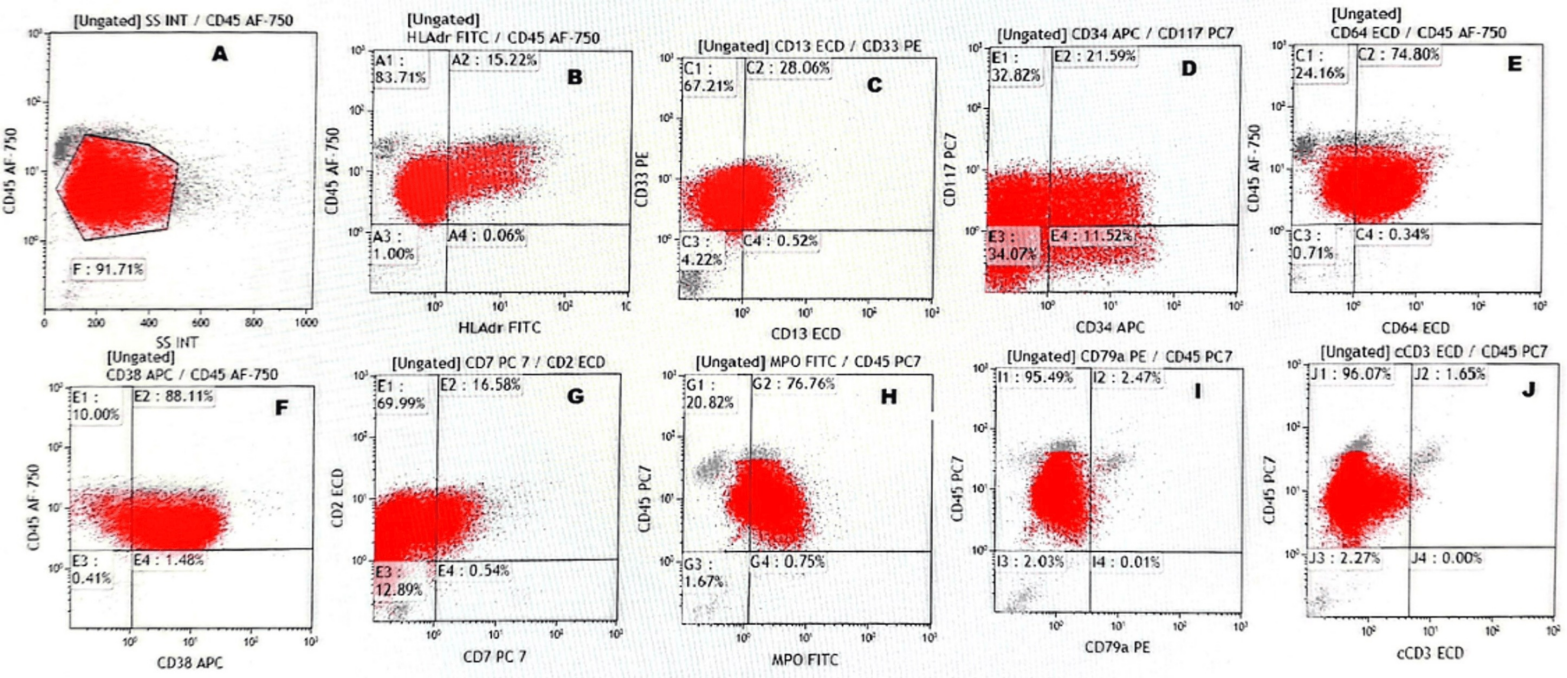
Flow cytometry dot plots showing immunophenotype of acute promyelocytic leukemia Gated population in red color (abnormal promyelocytes) expressing A: CD45 positive with moderate to high side scatter; B: HLA-DR positive; C: CD33 positive and CD13 dim positive; D: CD117 and CD34 positive; E: CD64 positive; F: CD38 positive; G: CD2 and CD7 positive (aberrant expression); H: MPO positive; I: CD79a negative; J: cCD3 negative

This diagnosis was further supported by findings on flow cytometry, karyotyping, and PCR for the AML panel. In flow cytometry, the blasts/abnormal promyelocyte population comprises 91% of total cellular events and is positive for CD45 with moderate to high side scatter, HLA-DR, CD34, CD33, CD117, CD13, CD64, CD11c, and MPO with aberrant expression of T cell markers CD2 and CD7. This population was negative for all other lineage-specific markers of B and T cell lineage (CD79a and cCD3, respectively) (Figure 5). 

Notably, this immunophenotypic profile was atypical for APL, which classically lacks expression of CD34 and HLA-DR. While aberrant CD2 expression has been reported in a subset of APL cases, concurrent aberrant expression of CD7 is less commonly described. Results of karyotyping showed t(15;17), and PCR for the AML panel also revealed PML-RARA. The coagulation profile of this patient was also deranged at the time of presentation, showing raised prothrombin time (PT) and fibrin degradation product (FDP) and reduced fibrinogen levels and activated partial thromboplastin time (Table 2). 

**Table 2:** Coagulation profile PT: prothrombin time; APTT: activated partial thromboplastin time; s: seconds

Coagulation Profile	Results	Reference Range
PT	13.8 s	9.9-12.7 s
Fibrin degradation product (FDP)	>20 ug/mL	<5.0 ug/mL
APTT	19.9 s	22.2-30.4 s
Fibrinogen	61.5 mg/dL	180-350 mg/dL

The patient was initiated on all-trans retinoic acid (ATRA), idarubicin, and steroids due to a high leukocyte count at presentation. The patient died a few days later due to treatment-related complications. She had complaints of a severe occipital headache and reduced vision. The CT scan showed an intracranial hemorrhage. She developed shortness of breath, pleural effusion, bilateral crepitations, and reduced oxygen saturation. She was placed on a ventilator. ATRA differentiation syndrome was suspected. Later, she developed acute kidney injury and electrolyte imbalance. The patient's family refused dialysis and further ventilator support due to financial reasons. The patient expired after being taken off the ventilator.

## Discussion

Post-cytotoxic therapy APL represents an infrequent subset of myeloid neoplasm post-cytotoxic therapy (MN-pCTs) and has been increasingly reported in breast cancer survivors as treatment outcomes and survival improve [9]. While secondary AML most often follows cytotoxic chemotherapy, post-cytotoxic therapy APL is distinctly uncommon, constituting approximately 1.7-5.8% of post-cytotoxic therapy AML cases [10]. Breast cancer represents the most frequently reported solid tumor preceding post-cytotoxic therapy APL, largely due to widespread use of anthracycline-based regimens, which induce balanced chromosomal translocations such as t(15;17) [9].

Published cases can be grouped into four main exposure patterns: multimodality chemotherapy with radiation and/or hormonal therapy; chemotherapy alone; chemotherapy and hormonal therapy; and radiation and/or hormonal therapy without preceding cytotoxic chemotherapy. Madabhavi et al. reported APL after chemotherapy alone with a latency period of around two years [9]. Ono et al. reported a case after radiation and hormonal therapy, about three and a half years later [10]. Mazumder et al. have reported a similar case after chemotherapy and hormonal therapy. The latency period of this case was around one and a half years [11]. However, according to the published case reports, post-cytotoxic therapy APL has been most frequently seen after multimodality therapy (chemotherapy plus hormonal therapy plus radiation therapy) [12-15]. The latency was around one and a half to three years.

The present case aligns with the category of hormonal plus radiation therapy without prior chemotherapy. The latency interval of approximately one year in our patient is among the shortest latency periods reported. Although radiation exposure has been associated with increased leukemia risk in a dose- and field-dependent manner, the absolute incidence remains low in breast cancer survivors treated with localized radiotherapy. Likewise, large cohort studies have not demonstrated a consistent association between hormonal therapy and secondary leukemia, underscoring the epidemiologic rarity of this presentation [10]. The exposure pattern associations and case reports, along with our case, are listed in detail in Table 3.

**Table 3:** Exposure patterns of post-cytotoxic therapy acute promyelocytic leukemia (APL) cases t (15;17): translocation 15;17; CR: complete remission; ATRA: all-trans retinoic acid; LHRH: luteinizing hormone-releasing hormone

Author / Year	Prior Therapy	Latency	Cytogenetics	Outcome
Madabhavi et al., 2015 [9]	Chemotherapy (Topoisomerase II inhibitors: Anthracyclines and Alkylating agents: Cyclophosphamide)	~23 months	t (15;17)	CR achieved
Ono et al., 2008 [10]	Radiation + Hormonal therapy (Gonadotropin-releasing hormone analog, tamoxifen, and medroxyprogesterone acetate (MPA))	~42 months	t (15;17)	Two CRs with ATRA + anthracycline
Mazumder et al., 2019 [11]	Chemotherapy (Topoisomerase II inhibitors and Alkylating agents: FEC regimen: 5-Fluorouracil, Epirubicin, Cyclophosphamide) + Hormonal (Aromatase inhibitor: Letrozole)	~18 months	t (15;17)	CR achieved
Mirili et al., 2018 [12]	Chemotherapy (Topoisomerase II inhibitors: Anthracyclines and Alkylating agents: Cyclophosphamide) + Radiation + Hormonal (Aromatase inhibitor)	~27 months	t (15;17)	CR achieved
Utsu et al., 2013 [13]	Chemotherapy (Topoisomerase II inhibitors: Anthracyclines and Alkylating agents: Cyclophosphamide) + Radiation + Hormonal therapy (LHRH analogs: leuprorelin and Tamoxifen)	~18 months	t (15;17)	CR achieved
Kara et al., 2011 [14]	Chemotherapy (Topoisomerase II inhibitors and Alkylating agents: CEF regimen: Cyclophosphamide, Epirubicin, and 5-Fluorouracil)+ Radiation + Hormonal therapy (Tamoxifen)	~36 months	t (15;17)	Not reported
Savooji et al., 2016 [15]	Chemotherapy (Topoisomerase II inhibitors: Anthracyclines and Alkylating agents: Cyclophosphamide) + Radiation + Hormonal (Aromatase inhibitor: Anastrozole)	Not reported	t (15;17)	CR achieved
Present Case	Hormonal therapy (LHRH analogs and Aromatase inhibitors: Letrozole) + Radiation + Bisphosphonate	~12 months	t (15;17)	Early mortality

From a prognostic standpoint, unlike other forms of post-cytotoxic therapy AML, APL demonstrates treatment responses and long-term outcomes comparable to de novo APL when managed promptly with ATRA-based regimens. Most published cases achieved complete remission, particularly within chemotherapy-associated clusters [16]. In contrast, our patient experienced early mortality despite rapid initiation of ATRA and anthracycline therapy for high-risk disease, reflecting the considerable risk of early hemorrhagic and differentiation-related complications that continue to influence early outcomes.

Taken together, the present case contributes epidemiological and clinical novelty by expanding the small cohort of post-cytotoxic therapy APL cases arising after hormonal and radiation therapy alone, with an unusually short latency interval and an adverse early outcome. As breast-conserving strategies and hormonal therapy become increasingly common, heightened vigilance for secondary hematologic malignancies is warranted, particularly in survivors presenting with unexplained cytopenias, bleeding, or coagulopathy.

## Conclusions

This case highlights a rare presentation of post-cytotoxic therapy APL following hormonal and radiation therapy for breast cancer without prior cytotoxic chemotherapy. The short latency interval and adverse early outcome underscore the importance of early recognition and prompt ATRA-based treatment in minimizing early mortality. With increasing survivorship in breast cancer patients, awareness of secondary leukemias such as post-cytotoxic therapy APL remains essential for timely diagnosis and management.

Compared with previously reported cases, this presentation is notable for the absence of cytotoxic chemotherapy exposure and for a relatively short latency period of approximately 12 months. While outcomes of post-cytotoxic therapy APL are generally reported to be comparable to de novo APL when treated promptly with ATRA-based regimens, early mortality remains a significant concern, particularly in patients presenting with high leukocyte counts and coagulopathy. This case expands the limited body of literature describing APL arising after hormonal therapy and radiotherapy alone and underscores the need for heightened vigilance for secondary hematologic malignancies in breast cancer survivors presenting with unexplained cytopenias or bleeding manifestations.
